# Prediction Models for Tinnitus Presence and the Impact of Tinnitus on Daily Life: A Systematic Review

**DOI:** 10.3390/jcm12020695

**Published:** 2023-01-16

**Authors:** Maaike M. Rademaker, Sebastiaan M. Meijers, Adriana L. Smit, Inge Stegeman

**Affiliations:** 1 Department of Otorhinolaryngology, Head and Neck Surgery, University Medical Center Utrecht, 3584 CX Utrecht, The Netherlands; 2 UMC Utrecht Brain Center, University Medical Center Utrecht, 3584 CX Utrecht, The Netherlands

**Keywords:** tinnitus, prediction model, tinnitus disorder, prediction model validation

## Abstract

The presence of tinnitus does not necessarily imply associated suffering. Prediction models on the impact of tinnitus on daily life could aid medical professionals to direct specific medical resources to those (groups of) tinnitus patients with specific levels of impact. Models of tinnitus presence could possibly identify risk factors for tinnitus. We systematically searched the PubMed and EMBASE databases for articles published up to January 2021. We included all studies that reported on multivariable prediction models for tinnitus presence or the impact of tinnitus on daily life. Twenty-one development studies were included, with a total of 31 prediction models. Seventeen studies made a prediction model for the impact of tinnitus on daily life, three studies made a prediction model for tinnitus presence and one study made models for both. The risk of bias was high and reporting was poor in all studies. The most used predictors in the final impact on daily life models were depression- or anxiety-associated questionnaire scores. Demographic predictors were most common in final presence models. No models were internally or externally validated. All published prediction models were poorly reported and had a high risk of bias. This hinders the usability of the current prediction models. Methodological guidance is available for the development and validation of prediction models. Researchers should consider the importance and clinical relevance of the models they develop and should consider validation of existing models before developing new ones.

## 1. Introduction

Prediction models are made to inform clinical decision making. They quantify the relative importance of findings, characteristics and different types of factors when evaluating an individual patient [1]. Over the past decade, there has been a steep increase in the number of prediction models in clinical research. Before it can be decided whether models on tinnitus prediction could be applied in clinical care and research, more clarity regarding the quality, performance and outcomes of these models is necessary.

Tinnitus can be described as the hearing of a phantom sound. The sheer presence of tinnitus does not necessarily imply associated suffering. Quality of life is severely reduced in 0.5–1% of the population due to tinnitus [2]. Because of this, recently two operational definitions have been proposed to distinguish between the two: tinnitus and tinnitus disorder [3]. To measure the impact of tinnitus on daily life multi-item questionnaires are used in clinical practice such as the Tinnitus Functional Index (TFI), the Tinnitus Handicap Inventory (THI) and the Tinnitus Questionnaire (TQ) or single-item questions [3,4,5,6].

Adequate prediction of the experience of tinnitus or the impact of tinnitus on daily life could be beneficial for preventive or therapeutic purposes. Prediction models on the impact of tinnitus on daily life could aid medical professionals to direct specific medical resources to those (groups of) tinnitus patients with specific levels of impact. Models on tinnitus presence could possibly identify risk factors for tinnitus. Through this, preventive measures could be taken to avoid the potential negative impact of tinnitus on daily life.

In prediction models, the patient specific value of each included factor is taken and combined to calculate risk estimates on the outcome for each individual. For adequate development of a clinically useful prediction model, three steps are needed. In the first step, the model is derived. This phase includes the identification of predictors, for which weights are obtained. Model validation is the second phase. During the development of a model, internal validation serves to assess and correct overfitting in the model. With external validation, the performance of the model is assessed in a different dataset. In the third and last phase, the model’s clinical impact is assessed by using the prediction rule as a decision rule [7]. In prognostic model development, it is advised that one should search, review, critically appraise and externally validate already existing prediction models before one starts to develop a new prediction model [7]. We aimed to systematically review the published prediction models of tinnitus presence and impact on daily life.

## 2. Materials and Methods

In this systematic review, we followed the Cochrane guidance for critical appraisal and data extraction for systematic reviews of prediction modelling studies (the CHARMS checklist) and the preferred reporting items for systematic reviews and meta-analyses (PRISMA) [8,9]. The protocol for this systematic review was registered at the international prospective register of systematic reviews (PROSPERO) with registration number CRD42021240493 [10].

### 2.1. Search Strategy

We searched the electronic literature databases of PubMed and EMBASE on the 21st of January 2021. The Ingui filter for finding studies on clinical prediction models was used in our search [11]. The search syntax can be found in Appendix A. In addition to the electronic database searches, reference lists were screened to identify additional studies. We searched for developmental as well as validation studies.

### 2.2. Study Selection/Eligibility Criteria

We included all studies that reported on multivariable prediction models. Multivariable models were defined as having two or more predictors included. Models were included when predicting the presence of tinnitus in adults or the effect of tinnitus on daily life. We included a broad range of outcomes to measure tinnitus-related effects on daily life. These included, but were not restricted to: tinnitus burden, tinnitus severity, tinnitus distress, tinnitus-associated quality of life, tinnitus-associated annoyance and tinnitus intrusiveness. These outcomes could be measured by using single-question and multiple-question questionnaires. We excluded letters to editors, reviews and animal studies. If articles reported multiple prediction models with a unique combination of predictors, we considered these as separate models.

We differentiated between articles reporting on the development and the external validation of studies. Articles were classified as developmental studies if the authors described the development of one or multiple models in their objectives or conclusions or if it was clear from other information (like information in the methods section) that a prediction model was developed in the study.

### 2.3. Screening Process

Two researchers (I.S., M.M.R.) independently screened the title and abstract of the articles for eligibility after removal of duplicates. Subsequently, the selected studies were reviewed for full text screening using predefined inclusion and exclusion criteria. Disagreements were resolved by discussion.

### 2.4. Data Extraction and Analysis

We created a data extraction form. This was based on the CHARMS checklist and previous research projects [9,12,13]. The following items were extracted from the included studies and included in the data extraction form: authors of the study, year of publication, journal of publication, the continent where the research was conducted, study design, study setting, instrument(s) used to measure the impact of tinnitus on daily life or tinnitus presence, the provided definition of tinnitus, percentage of patients with tinnitus in the study, mean impact of tinnitus on daily life measured with questionnaires or single questions, duration of tinnitus, number of research centres, number of participants, gender of the included patients, age of the included patients, horizon of prediction, number of predictor candidates, number of included predictor candidates in the final model, the number of predictor models, missing data, used statistical methods and the results of the prediction model. The data extraction form was triple checked by S.M.M.

### 2.5. Critical Appraisal (CAT)

The risk of bias (RoB) of the included studies was independently assessed by two researchers (M.M.R., I.S.) using the prediction model RoB assessment tool (PROBAST) [14]. The PROBAST tool consists of 20 signalling questions divided over four domains: participants, predictors, outcome and analysis. These domains were scored on RoB and applicability as low, high or unclear risk, based on the criteria that were provided by PROBAST [14]. PROBAST provided specific definitions for different domains to detect RoB. For example: the reasonable number of participants with a specific outcome relative to the number of candidate predictor candidates is defined as >20 (EPV >20) in model development studies. For the specific definition per domain and more explanation see: Moons et al. 2019: PROBAST: A tool to assess Risk of Bias and applicability of prediction model studies: Explanations and Elaboration [15]. Disagreements between the two researchers were solved by discussion.

### 2.6. Descriptive Analyses

The results of the data-extraction were summarized with descriptive statistics. No quantitative analyses were performed as this was beyond the scope of our study

## 3. Results

### 3.1. Search Results

**Figure 1:**
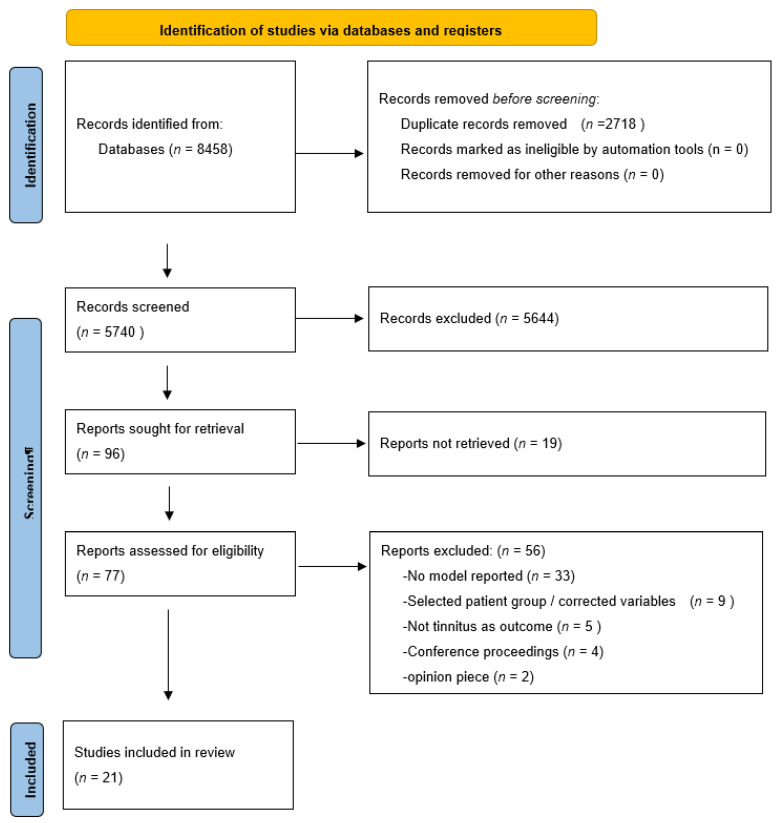
PRISMA flowchart.

Our search yielded 3241 hits on PubMed and 5217 hits on EMBASE. After deduplication (*n* = 2718), we screened 5740 articles on title and abstract. Of those, we read the full text of 73 articles. One study was screened after cross referencing and was not included in the final selection. Based on the predefined inclusion and exclusion criteria, we included 21 studies in this systematic review. Of those, 21 were developmental studies and 0 involved external validation of studies. (Figure 1: flowchart)

### 3.2. Developmental Studies

#### 3.2.1. Study Design and Study Populations

The 21 developmental studies were published between 1999 and 2021. Of these, 71% took place in Europe. Fourteen out of the 21 studies reported on one prediction model. Dawes et al., Andersson 2005 et al. and Beukes et al. reported on three models [16,17,18] and four studies reported on two models [19,20,21,22]. Four studies were retrospective cohort studies [20,23,24,25], two studies were prospective cohort studies [21,26] and 13 studies had a cross sectional design [16,17,18,19,22,27,28,29,30,31,32,33,34,35]. One had a nested case control design [36]. Twelve out of 21 studies were performed in a hospital setting at an outpatient clinic [17,18,20,22,23,24,25,26,29,30,32,35], seven studies were performed in the general population [16,19,21,27,28,31,34], one in a general practice setting and one in a combination of a hospital and the general population [33,36]. The number of participants per study varied between 44 and 168348. The reported mean age varied between 35.8 years and 69 years. The percentage of female participants ranged between 27.7% and 66.5%. The mean duration of tinnitus was reported in nine studies and ranged between 1.6 weeks and 12.5 years [17,18,20,22,24,25,26,29,32] (see Table 1).

#### 3.2.2. Risk of Bias

Based on the criteria that were provided by PROBAST [14], the overall RoB was judged to be high in all studies, mainly due to a high RoB in the analysis domain. No studies accounted for overfitting, underfitting or optimism. No studies reported on relevant model performance measures. The RoB in the participants, predictor and outcome domain was low. Ten studies reported on a reasonable number of participants with the outcome [16,17,19,21,27,28,29,31,33,36], and for four studies no information on this account was provided [25,26,34,35]. Eight studies did not handle missing data appropriately [16,18,20,23,25,27,29,31], and thirteen studies did not provide any information on missing data [17,19,21,22,24,26,28,30,32,33,34,35,36]. The applicability of the participants, predictor and outcome domain was judged to be low (see Table 2: CAT).

#### 3.2.3. Outcomes of Prediction Models

A total of 31 prediction models were described in the 21 included studies. Seventeen studies made a prediction model for the impact of tinnitus on daily life [17,18,19,20,22,23,24,25,26,27,29,30,31,32,33,34,35], three studies made a prediction model for tinnitus presence [21,28,36] and one study made models for both [16].

#### 3.2.4. Tinnitus Impact

The impact of tinnitus on daily life was assessed by using different multi-items in 13 studies [17,18,20,22,23,25,26,27,29,31,32,33,35]. The THI was used in eight studies [20,22,23,26,27,29,32,33]. The TQ was used by two studies [20,35] and the psychological distress scale of the TQ was used by one study [25]. The mini Tinnitus Questionnaire (mTQ) was used in one study [31]. One study used the Tinnitus Reaction Questionnaire (TRQ) [17]. One study used the Klockhoff and Lindblom classification of tinnitus severity scale [24]. Three studies used single-item questionnaires to measure the impact of tinnitus [16,19,30]. The questions and answer possibilities used are reported in Table 3.

The reported mean THI scores varied between 38.3 and 48.3 points. Bhatt also used the THI but did not report the mean THI score [27]. Instead, they reported that 88.5% of the patients had a THI score <16, whereas 8.6% had a score >18. Beukes et al. did not report the mean TFI score, but subdivided the TFI score into three categories demonstrating that 10% had a score below 25 (mild tinnitus), 30% had a score between 25 and 50 (significant tinnitus) and 60% had a sore above 50 (severe tinnitus) [18]. Wallhauser-Franke et al. categorized outcomes of scores using the mTQ: 37.6% had a total score of seven or lower, 49% had a total score between 8 and 18, and 13.4% had a total score of 19 or higher [31]. Andersson (2005) used the TRQ and reported a mean of 37.4 [17]. The studies using single-item questionnaires reported ‘bothersome tinnitus’ with different definitions in 9.1–30.9% of the cases [16,19,28].

##### Predictors of Tinnitus Impact

The number of candidate predictors reported in the included studies varied between two and 70 [16,17,18,19,20,22,23,24,25,26,27,29,30,31,32,33,34,35]. In three studies, the number and type of predictor candidates were not (clearly) reported and therefore the predictor candidates could not be extracted [25,26,34]. The five most common candidate predictors for tinnitus impact were: depression-related questionnaire scores (in 15 models), anxiety-related questionnaire scores (in 15 models), age (in 14 models), gender (in 9 models) and tinnitus duration (in 10 models) (Table 4/Appendix B).

The number of final model predictors for impact models differed between two and 13. In the prediction models on the impact on daily life, scores of questionnaires in which depressive symptoms (*n* = 12) were assessed or symptoms of anxiety (*n* = 8) were most commonly used. In addition, age (*n* = 5), gender (*n* = 3), alcohol use (*n* = 2), smoking (*n* = 2), occupational noise exposure (*n* = 2), music noise exposure (*n* = 2), tinnitus duration (*n* = 2) and tinnitus location (*n* = 1) were used.

##### Modelling Method and Prediction Horizon in Tinnitus Impact Models

Multiple different modelling methods were used: Multiple linear regression [17,23], Stepwise multiple regression [20,25,32], multivariable adjusted regression [19], hierarchical linear multiple regression [18], ordinal logit regression [26], discriminant function analysis [24], linear regression [27], multiple regression [35], stepwise multiple linear regression [22], multiple ordinary least square regression analysis [29], stepwise forward regression analysis [30,33], multiple logistic regression, backward elimination with complex sampling [34], binary stepwise logistic regression [31], and multinomial logistic regression [16]. Only the studies by Dawes et al., Holgers et al. and Langebach et al. had a reporting horizon of, respectively, 4.2 years, 18 and 6 months [16,25,30]. All other studies were cross-sectional designs.

##### Model Presentation and Predictive Performance in Tinnitus Impact Models

All except Andersson 1999 et al. [24] and Andersson 2005 et al. [17] presented a regression slope, and two studies also presented a intercept [18,30]. Overall model performance was reported by the proportion of variance (R^2^) in eleven studies [17,18,19,20,23,24,25,27,31,33]. Holgers et al. used a probability regression plot [30]. The other studies did not report about predictive performance [22,26,28,29,35,37]. (Table 5)

#### 3.2.5. Tinnitus Presence

Tinnitus presence was assessed with different questions. The questions and answer possibilities used are reported in Table 4. In Kostev et al., tinnitus presence was defined using the first International Classification of Diseases (ICP) diagnosis of tinnitus [36]. Patients with ICP diagnosed tinnitus were matched 1:1 with persons without tinnitus. (Table 6). The presence of tinnitus reported in the four studies varied between 17.3% and 59% [16,21,28,36].

##### Predictors of Tinnitus Presence

The number of candidate predictors reported in the included studies varied between 16 and 125 [16,21,28,36]. The most common candidate predictors for tinnitus presence were: Gender (in 5 models), age (in 3 models) and occupational or music noise exposure (both in 3 models). In the final models the most commonly used predictors were gender (*n* = 3) followed by age (*n* = 2). (Table 4/Appendix B).

##### Modelling Method and Prediction Horizon in Tinnitus Presence Models

Multiple different modelling methods were used: logistic hierarchical regression [28], multinomial logistic regression [16], Stepwise multivariate logistic regression [36], multinomial logit regression model [21]. Only the study of Dawes et al. had a prediction horizon of respectively 4.3 years [16]. The other studies had a cross-sectional design.

##### Model Presentation and Predictive Performance in Tinnitus Presence Models

All studies presented a regression slope. Couth et al. reported an intercept [28]. Overall model performance was reported by proportion of variance (R^2^) by two studies [16,28]. Moore et al. [21] used the Akaike Information Criterion [37]. Kostev et al. did not report their predictive performance [36]. (Table 6)

### 3.3. Validation Studies

Zero studies were internally validated.

## 4. Discussion

In this systematic review, we presented the published prediction models on tinnitus presence, and the impact of tinnitus on daily life. We identified 21 different studies with a total of 31 models. Of these 31 models, five reported on tinnitus presence and 26 on the impact of tinnitus on daily life. For models of tinnitus presence, the most common predictors were age, gender and smoking. For models in which the impact of tinnitus of daily life was predicted, scores of depression-associated questionnaires and anxiety-associated questionnaires were the most common. Model performance was mostly reported by using the proportion of variance (R^2^).

Despite the high number of developed models, the quality of prognostic modelling in tinnitus research is low. To date, regrettably, no models have been validated. Due to the lack of validation and impact analyses, the models cannot be used in clinical care. None of the included models were tested for calibration and discriminative performance [38]. Earlier studies showed that the discriminative and calibration abilities of models which are based on small datasets with simple statistical methods are generally poor. The use of categorized instead of continuous data further lowers that performance [39]. Therefore, it is necessary that sufficient statistical methods are used in the context of prediction modelling [38].

Van Royen et al. recently described the difficulties of model adaptation to clinical care. The authors described four reasons why the adaptation of prediction models can fail [7]. The first reason is that models do not fit a clinical purpose, for example when a model includes a patient population that does not correspondent with the patient population in the clinic. A second reason is that the model is not validated, or reporting is incomplete. As demonstrated in this manuscript, this is applicable for the present tinnitus models. This makes it difficult for clinicians and researchers to further develop and use the models. The third reason is that there are difficulties with the implementation—for example, when the model has no impact on decision making, or when local or national regulations are a hindrance to the implementation. The last reason is failed model adaption. Examples include non-useful or non-trusted predictions, or outdated models. Most of these reasons seem to fit the tinnitus literature, whereby the lack of validation, lack of fitness for purpose due to different opinions about outcome measures, included populations and poorly reported models seem to be most prominent.

Collaboration between different research groups can lead to less accumulation or repeating of studies [40]. An improvement in tinnitus prediction research might be to improve and intensify these collaborations. Currently, there is still room for improvement. For example, many similar predictor candidates were used by the different models, of which only a minority are used in the final model. We noticed that tinnitus-specific variables and variables on somatic comorbidities are most frequently used as predictor candidates. However, only in about 25% of the models were the tinnitus specific variables used in the final models. This is in contrast to demographic factors and somatic or psychological comorbidities. These groups of variables tend to end up in the final model in about 50%. This raises the question of whether or not we should continue researching the predictive value of tinnitus-specific variables or put the scope on other domains of characteristics. This review might serve as a base for future research groups to critically assess which predictor candidates or predictors they should use, to improve prediction models’ performance and their application in clinical practice. The focus could then be shifted towards model validation, rather than more model development studies.

Prediction models aim to provide guidance in clinical decision making, and should therefore be handled with care by those who develop the models. In all these stages of prediction model development, clinical knowledge about the setting, patients and pathways should be combined with the statistical and methodological know-how of model development. Therefore, we advise researchers to develop prediction models in a collaborative effort involving clinicians, statisticians and epidemiologists. The use of reporting tools can also be a helpful next step in improving tinnitus prediction modelling. Guidance can further be found in the PROBAST statement, which can help with identifying the risk of bias in prognostic studies, whereas the TRIPOD statement is suitable for guidance in reporting [14,41]. As demonstrated in our study, the majority of studies based their model on statistical methods. However, it is recommended to build models based on clinical expertise and previous literature, rather than making them purely data driven [42]. Other ideas to improve the quality of future research are the use of prospective, large, population-based studies, and the consequent use of similar, validated, outcome measures such as the TFI [3]. This would help compare prediction models in meta-analyses, and would ease external validation. This might help to create clinically applicable prediction models.

## 5. Conclusions

We identified 21 different studies, which report a total of 31 models on either the presence or the impact of tinnitus on daily life. All included models were in the development stage. The reporting of the models was found to be poor and the risk of bias high. No studies regarding model validation or risk assessment were found. Knowing the impact prediction models can have on clinical decision making as well as on directing future research and policy making, we need to improve the quality of our prediction research. Better reporting of methods, collaboration between research groups and disciplines could aid future prediction model development.

## Figures and Tables

**Table 1:** Study characteristics.

	Number of Models	Aims to Predict Tinnitus	Setting	Location	Design	Number of Centers	N = in Study	N = in Model	Age in Years Mean (SD, Range)	Gender (% Female)	Mean Duration of Tinnitus in Years (SD)
Aazh 2017 [23]	1	Impact	Outpatient clinic	Europe	RCS	1	184	148	69, (NR, NR)	NR	NR
Andersson 1999 [24]	1	Impact	Outpatient clinic	Europe	RCS	1	216	207	50.6 (13.8,14–77)	41%	7 (7.5)
Andersson 2005 [17]	3	Impact	Outpatient clinic	Europe	CSS	1	256	256	51 (13.6, 18–83)	43%	10.3 (13.6)
Basso 2020 [19]	2	Impact	General population	Europe	CSS	NA	7615	7615	35.8 (12.44, 11–84)	56.5%	NR
Beukes 2021 [18]	3	Impact	Outpatient clinic	Europe	CSS	3	326	326	55.5 (12.7, 22–83)	43%	10.3 (11.4)
Bhatt 2018 [27]	1	Impact	General population	North America	CSS	NA	678	289	NR (NR, 18–30)	66.5%	NR
Bruggeman 2016 [35]	1	Impact	Outpatient clinic	Europe	CSS	1	531	140	49 (13.29, 16–59)	53%	NR
Couth 2019 [28]	1	Presence	General population	Europe	CSS	NA	22,936	5727	53.9 (7.87, NR)	27.7%	NR
Dawes 2020 [16]	3	Impact and Presence	General population	Europe	CSS	NA	168,348	29,861 ▲	58.7 (7.58, NR)	47.2%	NR
Degeest 2016 [32]	1	Impact	Outpatient clinic	Europe	CSS	1	81	81	47.6 (14.4, 18–73)	35%	4.1 (6.2)
Han 2019 [22]	2	Impact	Outpatient clinic	Asia	CSS	1	248	248	Female: 55.8 (14.5, 20–82) Male: 52.2 (13.4, 20–82)	54%	Female: 29.1 (64.5) * Male: 42.1 (81.2) *
Hesser 2015 [29]	1	Impact	Outpatient clinic	Europe	CSS	1	362	316	59.6 (11.6, NR)	48%	12.5 (9.4)
Hoekstra 2014 [20]	2	Impact	Outpatient clinic	Europe	RCS	1	309	309	51 (NR, 17–82)	32.7%	7 (2-48) *
Holgers 2005 [30]	1	Impact	Outpatient clinic	Europe	CSS	1	127	127	Female 57 (16, NR) Male 52 (13, NR)	42.5%	NR
Kim 2015 [34]	1	Impact	General population	Asia	CSS	NA	19,290	4234	NR (NR,NR)	57%	NR
Kostev 2019 [36]	1	Presence	General practices	Europe	Nested case control	NA	37,692	37,692	57.5 (16.6, NR)	55.5%	NR
Langenbach 2005 [25]	1	Impact	Outpatient clinic	Europe	RCS	1	44	34	47.3 (NR, 19–78)	36.4%	1.6 (1.1) **
Moore 2017 [21]	2	Presence	General population	North America	PCS	NA	4950	4950	NR (NR, NR)	NR	NR
Strumilla 2017 [33]	1	Impact	Hospital & general population	Europe	CSS	1	212	212	48 (14.02, NR)	50.9%	NR
Unterrainer 2003 [26]	1	Impact	Outpatient clinic	Europe	PCS	2	149	149	51.6 (14.2, NR)	48.3%	711 (98.8) *
Wallhausser 2012 [31]	1	Impact	General population ^▼^	Europe	CSS	NA	4705	4705	58.6 (11.76, 18–94)	40.9%	NR

Symbols and abbreviations of Table 1: RCS= retrospective cohort study, PCS= prospective cohort study, CSS = cross sectional study NR = not reported * = in months, ** = in weeks ▲ = in the methods section *n*= 29,861 tinnitus sufferers were reported and *n* = 9751 patients with bothersome tinnitus. Age and gender are extracted from Table 2. ^▼^ = Survey sent to members of the German tinnitus association. In Table 3 *n* = 80,380 tinnitus sufferers were mentioned.

**Table 2:** Critical Appraisal of Topic (CAT).

	Signaling Questions	Aazh 2017 [23]	Andersson 1999 [24]	Andersson 2005 [17]	Basso 2020 [19]	Beukes 2021 [18]	Bhatt 2018 [27]	Bruggeman 2016 [35]	Couth 2019 [28]	Dawes 2020 [16]	Degeest 2016 [32]	Han 2019 [22]	Hesser 2015 [29]	Hoekstra 2014 [20]	Holgers 2005 [30]	Kim 2015 [34]	Kostev 2019 [36]	Langenbach 2005 [25]	Moore 2017 [21]	Strumilla 2017 [33]	Unterrainer 2003 [26]	Wallhausser 2012 [31]
1.Participant selection	1	YES	YES	YES	YES	YES	YES	YES	YES	YES	YES	YES	YES	YES	YES	YES	YES	YES	YES	YES	YES	YES
	2	YES	YES	YES	YES	YES	YES	YES	YES	YES	YES	YES	YES	YES	YES	YES	YES	YES	YES	YES	YES	YES
	Risk of bias	LOW	LOW	LOW	LOW	LOW	LOW	LOW	LOW	LOW	LOW	LOW	LOW	LOW	LOW	LOW	LOW	LOW	LOW	LOW	LOW	LOW
	1	YES	YES	YES	YES	YES	YES	YES	YES	YES	YES	YES	YES	YES	YES	YES	YES	YES	YES	YES	YES	YES
	Applicability	LOW	LOW	LOW	LOW	LOW	LOW	LOW	LOW	LOW	LOW	LOW	LOW	LOW	LOW	LOW	LOW	LOW	LOW	LOW	LOW	LOW
2.Predictors	1	YES	PY	YES	YES	YES	YES	YES	YES	YES	YES	YES	YES	YES	YES	YES	YES	YES	YES	YES	YES	YES
	2	YES	YES	YES	YES	YES	YES	YES	YES	YES	YES	YES	YES	YES	YES	YES	YES	YES	YES	YES	YES	YES
	3	NA	NA	NA	NA	NA	NA	NA	NA	NA	NA	NA	NA	NA	NA	NA	NA	NA	NA	NA	NA	NA
	Risk of bias	LOW	LOW	LOW	LOW	LOW	LOW	LOW	LOW	LOW	LOW	LOW	LOW	LOW	LOW	LOW	LOW	LOW	LOW	LOW	LOW	LOW
	Applicability	LOW	LOW	LOW	LOW	LOW	LOW	LOW	LOW	LOW	LOW	LOW	LOW	LOW	LOW	LOW	LOW	LOW	LOW	LOW	LOW	LOW
3.Outcome	1	YES	YES	YES	YES	YES	YES	YES	YES	YES	YES	YES	YES	YES	YES	YES	YES	YES	YES	YES	YES	YES
	2	YES	YES	YES	YES	YES	YES	YES	YES	YES	YES	YES	YES	YES	YES	YES	YES	YES	YES	YES	YES	YES
	3	YES	YES	YES	YES	YES	YES	YES	YES	YES	YES	YES	YES	YES	YES	YES	YES	YES	YES	YES	YES	YES
	4	YES	YES	YES	YES	YES	YES	YES	YES	YES	YES	YES	YES	YES	YES	YES	YES	YES	YES	YES	YES	YES
	5	YES	YES	YES	YES	YES	YES	YES	YES	YES	YES	YES	YES	YES	YES	YES	YES	YES	YES	YES	YES	YES
	6	YES	YES	YES	YES	YES	YES	YES	YES	YES	YES	YES	YES	YES	YES	YES	YES	YES	YES	YES	YES	YES
	Risk of bias	LOW	LOW	LOW	LOW	LOW	LOW	LOW	LOW	LOW	LOW	LOW	LOW	LOW	LOW	LOW	LOW	LOW	LOW	LOW	LOW	LOW
	Applicability	LOW	LOW	LOW	LOW	LOW	LOW	LOW	LOW	LOW	LOW	LOW	LOW	LOW	LOW	LOW	LOW	LOW	LOW	LOW	LOW	LOW
4.Analysis	1	NO	NO	YES	YES	NO	YES	NI	YES	YES	NO	NO	YES	NO	NO	NI	YES	NI	YES	YES	NI	YES
	2	YES	YES	YES	NO	YES	YES	YES	YES	YES	YES	YES	YES	YES	YES	NO	PY	YES	YES	YES	YES	NO
	3	NO	YES	PY	PY	YES	YES	NO	YES	YES	YES	YES	YES	YES	YES	YES	PY	YES	YES	YES	YES	YES
	4	NO	NI	NI	NI	NO	NO	NI	NI	NO	NI	NI	NO	NO	NI	NI	NI	NO	NI	NI	NI	NO
	5	YES	NO	NO	NO	NO	YES	YES	YES	YES	NO	YES	YES	NO	NO	NI	YES	NO	YES	YES	YES	YES
	6	NI	NI	NI	NI	NI	NI	NI	NI	NI	NI	NI	NI	NI	NI	YES	NI	NI	NI	NI	NI	NI
	7	NO	NO	NO	NO	NO	NO	NO	NO	NO	NO	NO	NO	NO	NO	NO	NO	NO	NO	NO	NO	NO
	8	NO	NO	NO	NO	NO	NO	NO	NO	NO	NO	NO	NO	NO	NO	NO	NO	NO	NO	NO	NO	NO
	9	NA	NA	Na	NA	NA	NA	NA	NA	NA	Na	NA	NA	NA	Na	NA	NA	NA	NA	NA	NA	NA
	Risk of Bias	High	High	High	High	High	high	High	high	High	high	High	High	High	High	High	High	High	high	High	High	High
Overall	Risk of Bias	High	High	High	High	High	High	High	High	High	High	High	High	High	High	High	High	High	High	High	High	High
	Applicabilty	LOW	LOW	LOW	LOW	LOW	LOW	LOW	LOW	LOW	LOW	LOW	LOW	LOW	LOW	LOW	LOW	LOW	LOW	LOW	LOW	LOW

Abbreviations: No information = NI, Probably YES = PY, Probably NO = PN. NA not applicable.

**Table 3:** Studies with impact of tinnitus on daily life as outcome.

		Outcome	Method Modelling	Mean Outcome of Measured Impact of Tinnitus on Daily Life	Prediction Horizon	# Predictor Candidates	# Predictors in Model
Aazh 2017 [23]		THI	Multiple linear regression	45.8 (23) ^1^	CS	11	7
Andersson 1999 [24]		Klockhoff and Lindbloms classification	Discriminant function analysis	grade I 5% Grade II 57% Grade III 38%	CS	21	4
Andersson 2005 [17]		TRQ (all)	Multiple linear regression	37.4 (26.8) ^2^	CS	8	8
		TRQ (Male)	Multiple linear regression	NR	CS	8	8
		TRQ (female)	Multiple linear regression	NR	CS	8	8
Basso 2020 [19]		Single question ^3^ (female)	Multivariable adjusted regression	9.1%	CS	37	13
		Single question ^3^ (male)	Multivariable adjusted regression	9.2%	CS	37	8
Beukes 2021 [18]		TFI	Hierarchical linear multiple regression	10% mild ^4^ 30% significant 60% severe	CS	23	3
Bhatt 2018 [27]		THI	Linear regression	88.5% THI < 16 8.7% THI > 18	CS	10	10
Bruggeman 2016 [35]		TQ	Multiple regression	34.73 (16.38) ^5^	CS	13	8
Dawes 2020 [16]		Single question ^6^	Multinomial logistic regression	5.8%	4.3 y (2–7)	13	13
Degeest 2016 [32]		THI	Stepwise multiple regression	44.2 (24.9)	CS	22	2
Han 2019 [22]	Female	THI (female)	Stepwise multiple linear regression	43 (25.9)	CS	9	2
	Male	THI (male)	Stepwise multiple linear regression	38.3 (25.9)	CS	9	3
Hesser 2015 [29]		THI	Multiple ordinary least square regression analysis	39.15 (22.2)	CS	7	7
Hoekstra 2014 [20]		TQ	Stepwise multiple regression	40 (17)	CS	28	4
		THI	Stepwise multiple regression	45 (23)	CS	28	5
Holgers 2005 [30]		Severe tinnitus ^7^	Stepwise forward regression analysis	24%	18 months	70	3
Kim 2015 [34]		Single question ^8^	Multiple logistic regression, backward elimination, complex sampling	30.9%	CS	NR	5
Langenbach 2005 [25]		Psychological distress of TQ scale	Multiple stepwise regression	NR	6 months	NR	3
Strumilla 2017 [33]		THI	Stepwise forward linear regression models	48.3 (22.54)	CS	2	2
Unterrainer 2003 [26]		THI	Ordinal logit regression	NR	CS	NR	9
Wallhausser 2012 [31]		Mini TQ	Binary stepwise logistic regression model	≤7: 37.6% 8–18: 49% ≥19: 13.4%	CS	15	8

Symbols and abbreviations: # = total number CS = cross sectional. ^1^ = mean of *n* = 178, model was made in *n* = 148. ^2^ = only provided for model including females and males. ^3^ = Question: “Is there a constant ringing in the ears or do you have any other bothersome sound in the ears (tinnitus)? Answer: Constant and bothersome: “All the time, the sound is very bothersome” or Intermittent and non-bothersome: “Sometimes, but the sound doesn’t bother me”. ^4^ = mild = 0–25 points, significant 25–50 points, severe = 50 or more points. ^5^ = of all participants, model in *n* = 140. ^6^ = How much do these noises worry, annoy or upset you when they are at their worst?’; severely, moderately, slightly or not at all. In this analysis, ‘bothersome’ tinnitus was identified on the basis of responses of either ‘moderately’ or ‘severely’. ^7^ = Severe tinnitus suffering (STS) refers to patients who fulfilled the following criteria: (1) Absence from work more than one consecutive month, (2) more than three visits to the therapist or the audiological physician. The STS and non-STS patient groups were compared. ^8^ = Have you heard any ringing, buzzing, roaring, or hissing sounds without an external acoustic source in the past year? If yes: do these sounds bother you? No, a little annoying, and very annoying.

**Table 4:** Most frequently used predictor candidates and included predictors.

	Predictor Candidates		In Final Model	
Predictor Category	# Predictor Candidates in Tinnitus Presence Models	# Predictor Candidates in Model on Tinnitus Impact on Daily Life	# Used in Tinnitus Presence Models	# Used in Models on Tinnitus Impact on Daily Life
**Demographic**				
age	4	15	2	5
Gender	4	9	3	3
**Risk factors**				
Alcohol use	1	5	1	2
Smoking	1	5	2	2
**Noise exposure**				
Occupational noise exposure	3	2	1	2
Music noise exposure	2	2	1	2
**Tinnitus specific**				
Duration	0	10	0	2
Location	0	9	0	1
**Depression**				
Depression questionnaires combined	0	15	0	12
**Anxiety**				
Anxiety questionnaires combined	0	12	0	8

# = total number.

**Table 5:** Overall reported performance measures.

		Prediction Models on Tinnitus Impact on Daily Life	Prediction Models on Tinnitus Presence
Overall performance measures	R^2^	11 [16,17,18,19,20,23,24,25,27,29,32]	2 [16,32]
	Other	1 [30]	1 [21]
	Any	-	
Discrimination and calibration measures	C statistic/AUC	-	
	Other	-	
	Hosmer Lemeshow	-	
	Other	-	
Internal validation		-	

Abbreviations: R^2^ = R-squared; AUC = Area under the receiver operating characteristic curve.

**Table 6:** Studies with tinnitus presence as an outcome.

	Outcome	Method Modelling	Presence	Prediction Horizon	# Predictor Candidates	# Predictors in Model
Couth 2019 [28]	Single question ^1^	Logistic hierarchical regression	17.29%	CS	16	16
Dawes 2020 [16]	Single question ^2^	Multinomial logistic regression	17.7%	4.3 y (2–7)	13	13
Kostev 2019 [36]	ICP diagnosis of tinnitus ^3^	Stepwise multivariate logistic regression	1:1 matched cohort with 18,846 tinnitus patients	CS	125	20
Moore 2017 [21]	Tinnitus frequency (rate of occurrence) ^4^	Multinomial logit regression models (se regression)	59%	CS	12	6

Abbreviations and symbols: # = total number. CS = cross sectional. ^1^ ‘Do you get or have you had noises (such as ringing or buzzing) in your head or in one or both ears that last more than 5 min at a time?” (a) Yes, now, most or all of the time; (b) Yes, now, a lot of the time; (c) Yes, now, some of the time; (d) Yes, but not now, but have in the past; (e) No, never; (f) Do not know; or (g) Prefer not to answer. The presence of tinnitus was characterized by participants currently having symptoms at least “now some of the time. ^2^ ‘Do you get or have you had noises (such as ringing or buzzing) in your head, or in one or both ears, that last for more than five min at a time?’ yes most of the time’, ‘yes a lot of the time’ or ‘yes some of the time. ^3^ Patients who had received a first tinnitus diagnosis (International Classification of Diseases, 10th revision [ICD-10]: H93.1). ^4^ How often nowadays do you get tinnitus (noises such as ringing or buzzing in your heard or ears) that lasts for more than.

## Appendix A

**Table A1:** Search strategy.

PubMed	(“Tinnitus”[Mesh] OR Tinnitus [tiab])
AND	((“Risk Factors”[Mesh] OR “Predictive Value of Tests”[Mesh] OR prediction model*[tiab] OR prediction rule*[tiab] OR decision support*[tiab] OR predictive model*[tiab] OR risk prediction*[tiab] OR risk scoring system*[tiab] OR scoring scheme*[tiab] OR risk assessment*[tiab] OR risk appraisal*[tiab] OR risk assessor*[tiab] OR risk calculation*[tiab] OR risk factor*[tiab] OR predict*[tiab] OR scoring system*[tiab]) OR ((Validat*[tiab] OR Predict*[tiab] OR Rule*[tiab]) OR (Predict*[tiab] AND (Risk*[tiab] OR Model*[tiab])) OR ((Criteria[tiab] OR Scor*[tiab]) AND (Predict*[tiab] OR Model*[tiab] OR Decision*[tiab] OR Prognos*[tiab]) OR (Decision*[tiab] AND (Model*[tiab] OR logistic models[mesh])) OR (Prognostic[tiab] AND (Criteria[tiab] OR Scor*[tiab] OR Model*[tiab])))) OR ((“Discrimination”[tiab] OR “Discriminate”[tiab] OR “c-statistic”[tiab] OR “c statistic”[tiab] OR “Area under the curve”[tiab] OR “AUC”[tiab] OR “Calibration”[tiab] OR “Algorithm”[tiab])))) OR (((tinnitus[Title/Abstract]) OR (tinnitus[MeSH Terms])) AND ((characterist*[Title/Abstract]) OR (risk*[Title/Abstract])))
EMBASE	‘Tinnitus’/exp OR Tinnitus :ti,ab,kw
AND	(‘risk factor’/exp OR ‘risk assessment’/exp OR ‘predictive value’/exp OR ‘prediction’/exp OR prediction model*:ti,ab,kw OR prediction rule*:ti,ab,kw OR decision support*:ti,ab,kw OR predictive model*:ti,ab,kw OR risk prediction*:ti,ab,kw OR risk scoring system*:ti,ab,kw OR scoring scheme*:ti,ab,kw OR risk assessment*:ti,ab,kw OR risk appraisal*:ti,ab,kw OR risk assessor*:ti,ab,kw OR risk calculation*:ti,ab,kw OR risk factor*:ti,ab,kw OR predict*:ti,ab,kw) OR (validat*:ti,ab,kw OR predict*:ti,ab,kw OR rule*:ti,ab,kw OR (predict*:ti,ab,kw AND (risk*:ti,ab,kw OR model*:ti,ab,kw)) OR ((criteria:ti,ab,kw OR scor*:ti,ab,kw) AND (predict*:ti,ab,kw OR model*:ti,ab,kw OR decision*:ti,ab,kw OR prognos*:ti,ab,kw)) OR (decision*:ti,ab,kw AND (model*:ti,ab,kw OR logistic) AND ‘models’/exp) OR (prognostic:ti,ab,kw AND (criteria:ti,ab,kw OR scor*:ti,ab,kw OR model*:ti,ab,kw)
OR	((Tinnitus:ti,ab,kw OR ‘tinnitus’/exp) AND (characterist*:ti,ab,kw OR risk*:ti,ab,kw))

## Appendix B

**Table A2:** Used predictor candidates per study.

Predictor Categories			Tinnitus Presence Studies		Impact on Daily Life Studies	
Demographic		# used as predictor candidate for different (final) models	candidates	In final model	candidates	In final model
	Age	6	Couth 2019, Moore (2×), Dawes (1×)	Couth 2019, Dawes (1×)	Aazh, Basso 2020 (2×), Beukes 2020 (3×), Degeest 2016, Han 2019 (2×), Hesser 2016, Hoekstra 2014 (2×), Wallhauser 2012, Dawes (1×), Holgers 2005	Basso 2020 (2×), Hesser 2016, Kim 2015, Dawes (1×)
	Gender	5	Couth 2019, Moore (2×), Dawes (1×)	Couth 2019, Dawes (1×)	Beukes 2020 (3×), Bhatt 2018 (1×), Degeest 2016, Hoekstra 2014 (2×), Wallhauser 2012, Dawes (1×)	Bhatt 2018 (1×), Kim 2015, Dawes (1×)
	Ethnicity	2	Couth 2019	Couth 2019	Bhatt 2018 (1×),	Bhatt 2018 (1×)
	SES	2	Dawes (1×)	Dawes (1×)	Dawes (1×)	Dawes (1×)
	Townsend Quartiel	1	Couth 2019	Couth 2019		
	Marital Status	1			Basso 2020 (2×)	Bruggemann 2016
	Employment	1			Basso 2020 (2×), Hoekstra 2014 (2×)	Basso
	Industry type (vs. finance)					
	Agricultural	1	Couth 2019	Couth 2019		
	construction	1	Couth 2019	Couth 2019		
	Music	1	Couth 2019	Couth 2019		
	Income level				Holgers 2005	
	Educational level	2			Basso 2020 (2×), Hoekstra 2014 (2×), Holgers 2005	Basso 2020 (2×), Hoekstra 2014 (1×)
Risk Factors	Alcohol use	2	Couth 2019	Couth 2019	Basso 2020 (2×), Dawes (2×), Holgers 2005	Dawes (2×)
	Smoking	3	Couth 2019	Couth 2019	Basso 2020 (2×), Bhatt 2018 (1×), Dawes (1×), Holgers 2005	Bhatt 2018 (1×), Dawes (1×)
	Snus use	1			Basso 2020 (2×)	
	Drug use	1			Basso 2020 (2×)	
	Ototoxic medication	3	Couth 2019, Dawes (1×)	Couth 2019, Dawes (1×)	Dawes (1×)	Dawes (1×)
Noise exposure	Loud noise exposure	0			Beukes 2020 (3×)	
	Occupational noise exposure	3	Couth 2019, Moore (2×)	Couth 2019	Dawes (2×)	Dawes (2×)
	Music noise exposure	3	Couth 2019, Moore (2×)	Couth 2019	Dawes (2×)	Dawes (2×)
Tinnitus specific						
	Pitch	1			Degeest 2016	Andersson 1999
	Pitch (VAS)	1			Hoekstra 2014 (2×)	Unterrainer 2003
	Tinnitus loudness	2			Bhatt 2018 (1×), Degeest 2016, Hesser 2016	Bhatt 2018 (1×), Hesser 2016
	Loudness VAS	4			Aazh, Han 2019 (2×), Hoekstra 2014 (2×)	Aazh (1×), Hoekstra 2014 (2×), Unterrainer 2003
	Duration	2			Beukes 2020 (3×), Bhatt 2018 (1×), Degeest 2016, Han 2019 (2×), Hoekstra 2014 (2×), Wallhauser 2012	Bhatt 2018 (1×), Bruggemann 2016
	Variability in pitch and loudness	1			Hoekstra 2014 (2×)	Hoekstra 2014 (1×)
	How often is the tinnitus heard				Beukes 2020 (3×)	
	Complex sound	1			Hesser 2016	Hesser 2016
	Family history of tinnitus	0			Degeest 2016	
	Pulsatile	0			Beukes 2020 (3×)	
	Initial onset (gradual/abrupt)	0			Degeest 2016, Hoekstra (2×) Wallhauser 2012	
	Location	1			Beukes 2020 (3×), Degeest 2016, Han 2019, (2×) Hoekstra (2×), Walhauser 2012	Wallhauser 2012
	Age at onset	0			Hoekstra 2014 (2×)	
	Type of tinnitus	0			Beukes 2020 (3×), Hoekstra 2014 (2×)	
	Number of sounds	0			Hoekstra 2014 (2×)	
	Tinnitus awareness	2			Degeest 2016, Hoekstra 2014 (2×)	Hoekstra 2014 (2×)
	Permanent awerenss	1			Wallhauser 2012	Wallhauser 2012
	Tinitus awareness vas	0			Han 2019 (2×)	
	Tinnitus presence (vas)	0			Hoekstra 2014 (2×)	
	Tinnitus annoyance (VAS)	2			Aazh, Han 2019 (2×)	Aazh, Han 2019 (1×)
	Tinnitus effect on life (VAS)	2			Aazh, Han 2019 (2×)	Aazh, Han 2019 (1×)
	Tinnitus Acceptance questionnaire	1			Hesser 2016	Hesser 2016
	Change in perception over time	0			Hoekstra 2014 (2×)	
	Tinnitus changed significantly	0			Beukes 2020 (3×)	
	Working less because of tinnitus	0			Beukes 2020 (3×)	
	Tolerance in relation to onset	1				Andersson 1999
Influence on tinnitus	masking of tinnitus by environmental/external sounds	0			Degeest 2016, Hoekstra 2014 (2×)	
	Influence of head and neck movement	0			Degeest 2016	
	sounds distract or mask tinnitus	0			Beukes 2020 (3×)	
	Somatosensory modulation	0			Hoekstra 2014 (2×)	
Tinnitus treatment	medication to help tinnitus or comorbidities	0			Beukes 2020 (3×)	
	Previous tinnitus treatment	0			Beukes 2020 (3×)	
Hearing loss	Hearing ability	1			Basso 2020 (2×)	Basso 2020 (2×)
	Hearing related difficulties	2	Dawes (1×)	Dawes (1×)	Degeest 2016, Wallhauser 2012, Dawes (1×)	Dawes (1×)
	Hearing related difficulties in social situations	2			Basso 2020 (2×)	Basso 2020 (2×)
	Self-reported hearing loss	1			Beukes 2020 (3×), Bhatt 2018 (1×)	Bhatt 2018 (1×)
	Presence of hearing loss	0			Han 2019 (2×)	
	Hearing disability (HHIA-S)	2			Beukes 2020 (3×)	Beukes 2020 (2×)
	Hearing aids	1			Beukes 2020 (3×), Degeest 2016, Wallhauser 2012	Wallhauser 2012
Hyperacusis	Hyperacusis subjective	0			Hoekstra 2014 (2×)	
	Hyperacusis Questionnaire	2			Aazh, Beukes 2020 (3×), Degeest 2016	Aazh, DeGeest 2016
	Subjective noise tolerance	0			Degeest 2016	
	Sound sensitivity	1			Hesser 2016	Hesser 2016
	Sound level tolerance	1			Bhatt 2018 (1×)	Bhatt 2018 (1×)
	Distortion of sound	0			Hoekstra 2014 (2×)	
Audiological measures						
	PTA	0			DeGeest 2016, Hoekstra 2014 (2×)	
	PTA worse ear	0			Aahz	
	PTA better ear	0			Aahz	
	PTA (0.5,1,2 Hz) right ear	0			Holgers 2005	
	PTA (0.5,1,2 Hz) left ear	0			Holgers 2005	
	PTA (0.5,1,2 Hz) both ears	0			Holgers 2005	
	PTA (2,4,6 Hz) right ear	0			Holgers 2005	
	PTA (2,4,6 Hz) left ear	0			Holgers 2005	
	PTA (2,4,6 Hz) both ears	0			Holgers 2005	
	Hearing loss	1				Kim 2015
Speech perception	Speech in noise right ear	0			Holgers 2005	
	Speech in noise left ear	0			Holgers 2005	
	Speech in noise both ears	0			Holgers 2005	
	SRT better ear	2	Dawes (1×)	Dawes (1×)	Dawes (1×)	Dawes (1×)
Loudness/Hyperacusis tests	average ULL in ear with lowest ULL	0			Aazh	
	Loudness discomfort Levels	0			Degeest 2016, Hoekstra 2014 (2×)	
Masking	MMI white noise	0			Degeest 2016	
	MMI narrow band noise					
	Residual inhibition	0			Degeest 2016, Hoekstra 2014 (2×)	
Tinnitus						
	Loudness matchting	0			DeGeest 2016, Hoekstra 2014 (2×)	
	Pitch matching	0			Degeest 2016, Hoekstra 2014 (2×)	
	Audiometric maskability	0			Hoekstra 2014 (2×)	
	Minimal masking levels	1			Degeest 2016, Hoekstra 2014 (2×)	Andersson 1999
Comorbidities	Sleep					
	Poor sleep quality	2			Basso 2020 (2×)	Basso 2020 (2×)
	Sleep problems	1			Wallhauser 2012	Wallhauser 2012
	Insomnia (ISIS)	3			Aazh (1×), Beukes 2020 (3×)	Aazh, Beukes 2020 (2×)
	Sleep disturbances	0			Basso 2020 (2×)	
	Initial insomnia (van structrd tnitus interview)	1				Langebach 2005 (1×)
Cardiovascular	Cardiovascular disease	2	Couth 2019	Couth 2019	Basso 2020 (2×)	Basso 2020 (1×)
	Hypertension	0	Couth 2019	Couth 2019	Basso 2020 (2×)	
	Hyperlipedemia	2	Couth 2019	Couth 2019	Basso 2020 (2×)	Kim 2015
	Diabetes	0	Couth 2019	Couth 2019	Basso 2020 (2×)	
	BMI	1	Couth 2019	Couth 2019	Holgers 2005	
Pain						
	Pain complaints	0			Hoekstra 2014 (2×)	
	Chronic pain	1			Wallhauser 2012	Wallhauser 2012
	Fibromyalgia	1			Basso 2020 (2×)	Basso 2020 (1×)
	Chronic shoulder pain	2			Basso 2020 (2×)	Basso 2020 (2×)
Ear						
	Vertigo	0			Hoekstra 2014 (2×)	
	Otalgia	0			Hoekstra 2014 (2×)	
	Ear fullness	0			Hoekstra 2014 (2×)	
	Recurring ear infections	1			Bhatt 2018 (1×)	Bhatt 2018 (1×)
	Dizziness	0			Wallhauser 2012	
	Morbus Meniere	1			Basso 2020 (2×)	Basso 2020 (1×)
Neurological	Epilepsy	1			Basso 2020 (2×)	Basso 2020 (1×)
	Multiple sclerosis	0			Basso 2020 (2×)	
Other	Asthma	0			Basso 2020 (2×)	
	Thyroid disease	1			Basso 2020 (2×)	Basso 2020 (1×)
	Metabolic risk	2	Dawes (1×)	Dawes (1×)	Dawes (1×)	Dawes (1×)
	Rheumatoid arthritis	0			Basso 2020 (2×)	
	Systematic lupus erythematosus	0			Basso 2020 (2×)	
	Somatic complaints	1			Hoekstra 2014 (2×)	Hoekstra 2014 (1×)
	Migraine	0			Basso 2020 (2×)	
	Osteoarthritis	1			Basso 2020 (2×)	Basso 2020 (1×)
	Somatic comorbidities	0			Wallhauser 2012	
	Health history	1			Bhatt 2018 (1×)	Bhatt 2018 (1×)
	Comorbidity	1				Unterrainer 2003
Comorbidities psychological						
	Depression	0			Basso 2020 (2×)	
	HADS-D	5			Aazh, Andersson 2005 (3×), Hesser 2016	Aazh, Andersson 2005 (3×), Hesser 2016
	BDI	2			Han 2019 (2×)	Han 2019 (2×)
	PHQ9/15	2			Beukes 2020 (3×), Wallhauser 2012	Beukes 2020 (1×), Wallhauser 2012
	Algemeines depression skala (ADS)	1				Unterrainer 2003
	Self reported depression and/or anxiety	2			Hoekstra 2014 (2×)	Hoekstra 2014 (2×),
Anxiety	Hads A	5			Aazh, Andersson 2005 (3×), Hesser 2016	Aazh, Andersson 2005 (3×), Hesser 2016
	Generalized anxiety syndrome	1			Basso 2020 (2×)	Basso 2020 (1×)
	GAD	1			Beukes 2020 (3×), Wallhauser 2012	Wallhauser 2012
	Panic disorder	0			Basso 2020 (2×)	
	Agoraphobia	0			Basso 2020 (2×)	
	Social anxiety	0			Basso 2020 (2×)	
	Anxiety (SCL-90-R)	1				Langebach 2005 (1×)
Stress	PTSS	0			Basso 2020 (2×)	
	Perceived Stress Questionnaire	1				Bruggemann 2016
	Bepsi-K	1			Han 2019 (2×)	Han 2019 (1×)
	traumatic/stressful experiences	0			Basso 2020 (2×)	
	Stress	1				Kim 2015
Other	Burnout	1			Basso 2020 (2×)	Basso 2020 (1×)
	Bipolar	0			Basso 2020 (2×)	
	Obsessive compulsive disorder	0			Basso 2020 (2×)	
	PHQ15	0			Wallhauser 2012	
	Diagnosed with a psychological condition	0			Beukes 2020 (3×)	
	‘Avoidance of situations because of tinnitus’	1				Andersson 1999
QoL	Satisfaction of life (SWLQ)	0			Beukes 2020 (3×)	
Cognition	cognitive failures (CFq)	0			Beukes 2020 (3×)	
Other	Noise dose	1			Bhatt 2018 (1×)	Bhatt 2018 (1×)
	Physical activity	3	Couth 2019, Dawes (1×)	Couth 2019, Dawes (1×)	Dawes (1×)	Dawes (1×)
	Neuroticism	1			Dawes (1×)	Dawes (1×)
Personality	Life satisfaction (freiburger personalitatinvntar)	1				Langebach 2005 (1×)
	Five Big Personality dimensions scale	1			Strumila 2017	Strumila 2017
Internal Locus of control		1				Unterrainer 2003
external locus of control		1				Unterrainer 2003
Fatalistic externality		1				Unterrainer 2003
	Perception of illeness	1				Unterrainer 2003
Perfectionism	concern over mistake	3			Andersson 2005 (3×)	Andersson 2005 (3×)
	personal standards	3			Andersson 2005 (3×)	Andersson 2005 (3×)
	parental expectations	3			Andersson 2005 (3×)	Andersson 2005 (3×)
	parrental criticism	3			Andersson 2005 (3×)	Andersson 2005 (3×)
	doubts about action	3			Andersson 2005 (3×)	Andersson 2005 (3×)
	organisation	3			Andersson 2005 (3×)	Andersson 2005 (3×)
TSQ	1 how much does tinnitus reduce the quality of life overall	0			Holgers 2005	
	2. when you are in a quiet environment, but not trying to sleep, how much discomfort does your tinnitus cause	0			Holgers 2005	
	3. how often do you notice tinnitus during your waking hours	0			Holgers 2005	
	4. how often does tinnitus impair your concentratio, for example when reading	0			Holgers 2005	
	5. how often is it difficult for you to go to sleep, and get back to sleep, due to tinnitus?	0			Holgers 2005	
	how often can you surpress or forget your tinnitus by some acitivy, for example watching TV or talking to somebody?	0			Holgers 2005	
	7. if you are exposed to every day sounds, how easily do these sound reduce or drown you rtinnitus	0			Holgers 2005	
	8. how often does tinnitus make you feel anxious or worried?	0			Holgers 2005	
	9. how often does tinnitus makeyou feel tense or irritable?	0			Holgers 2005	
	10. how often does tinnitus make you feel depressed and miserable?	0			Holgers 2005	
Nottingham health profile (NHP)	emotional distrubances	0			Holgers 2005	
	sleep distrubances	0			Holgers 2005	
	energy	0			Holgers 2005	
	pain	0			Holgers 2005	
	physical mobility	0			Holgers 2005	
	social isolation	0			Holgers 2005	
NHP Emotional disturbances	I feel that life is not worth living	1			Holgers 2005	Holgers 2005
	Worry is keeping me awake at night	0			Holgers 2005	
	I feel as if im losing control	0			Holgers 2005	
	Things are getting me down	0			Holgers 2005	
	I’ve forgotten what it’s like to enjoy myself	0			Holgers 2005	
	I wake up feeling depressed	0			Holgers 2005	
	I lose my temper easily these days	0			Holgers 2005	
	The days seem to drag	0			Holgers 2005	
	I’m feeling on edge	0			Holgers 2005	
NHP sleep disturbances	I lie awake for most of the night	0			Holgers 2005	
	I take tablets to help me sleep	0			Holgers 2005	
	I sleep badly at night	1			Holgers 2005	Holgers 2005
	It takes me a long time to get to sleep	0			Holgers 2005	
	I’m waking up in the early hours of the morning	0			Holgers 2005	
NHP energy	Everything is an effort	0			Holgers 2005	
	I’m tired all the time	0			Holgers 2005	
	I soon run out of energy	0			Holgers 2005	
NHP Pain	I’m in constant pain	0			Holgers 2005	
	I have unearable pain	0			Holgers 2005	
	I have pain at night	0			Holgers 2005	
	I’m in pain when I walk	0			Holgers 2005	
	I find it painful to change position	0			Holgers 2005	
	I’m in pain when I’m sitting	0			Holgers 2005	
	I’m in pain when I’m standing	0			Holgers 2005	
	I’m in pain when going up and down stairs	0			Holgers 2005	
NHP Physical mobility	I am unable to walk at all	0			Holgers 2005	
	I find it hard to dress myself	0			Holgers 2005	
	I need help to walk about outside	0			Holgers 2005	
	I can only walk about indoors	0			Holgers 2005	
	I find it hard to bend	0			Holgers 2005	
	I have trouble getting up and down stairs	0			Holgers 2005	
	I find it hard to stand for long	0			Holgers 2005	
	I find it hard to reach for things	0			Holgers 2005	Holgers 2005
NHP social isolation	I feel I am a burden to people	0			Holgers 2005	
	I feel lonely	0			Holgers 2005	
	I feel there is nobody I am close to	0			Holgers 2005	
	I’m finding it hard to make contact with people	0			Holgers 2005	
	I’m finding it hard to get on with people	0			Holgers 2005	
International classification of disease 10th revision (ICD-10)	Diseases of the ear (diseases of middle ear and mastoid) [H65–H75]	0	Kostev 2019			
	H65 Nonsuppurative otitis media	1	Kostev 2019	Kostev 2019		
	H66 Suppurative and unspecified otitis media	1	Kostev 2019	Kostev 2019		
	H68 Eustachian salpingitis and obstruction	1	Kostev 2019	Kostev 2019		
	Diseases of inner ear [H80–H83]	0	Kostev 2019			
	H81.0 Menieres disease	1	Kostev 2019	Kostev 2019		
	H81.1 Benign paroxysmal vertigo	1	Kostev 2019	Kostev 2019		
	H81.2 Vestibular neuronitis	1	Kostev 2019	Kostev 2019		
	H81.9 Disorder of vestibular function, unspecified	1	Kostev 2019	Kostev 2019		
	Other disorders of ear [H90–H95]	0	Kostev 2019			
	H91.9 presbycusis	1	Kostev 2019	Kostev 2019		
	H92 Otalgia and effusion of thee ar	1	Kostev 2019	Kostev 2019		
	Diseases of the upper respiratory tract (J30–J39)	0	Kostev 2019			
	J30 Allergic rhinitis	1	Kostev 2019	Kostev 2019		
	J31 Chronic rhinitis	1	Kostev 2019	Kostev 2019		
	J32 Chronic sinusitis	1	Kostev 2019	Kostev 2019		
	Mental disorders (organic, including symptomatic, mental disorders [F00–F09]	0	Kostev 2019			
	Mood [affective] disorders [F30–F39]	0	Kostev 2019			
	F32, F33 Depression	1	Kostev 2019	Kostev 2019		
	Neurotic, stress-related, and somatoform disorders [F40–F48]	0	Kostev 2019			
	F41 Anxiety disorder	1	Kostev 2019	Kostev 2019		
	F43 Reaction to severe stress, and adjustment disorders	1	Kostev 2019	Kostev 2019		
	F45 somatoform disorders	1	Kostev 2019	Kostev 2019		
	Diseases of the nervous system (extrapyramidal and movement disorders [G20–G26]	0	Kostev 2019			
	Other degenerative diseases of the nervous system [G30–G32]	0	Kostev 2019			
	Demyelinating diseases of the central nervous system [G35–G37]	0	Kostev 2019			
	Episodic and paroxysmal disorders [G40–G47]	0	Kostev 2019			
	G43 migraine	1	Kostev 2019	Kostev 2019		
	Endocrine diseases (disorders of the thyroid gland [E00–E07]	0	Kostev 2019			
	Diabetes mellitus [E10–E14]	0	Kostev 2019			
	Diseases of the circulatory system (hypertensive diseases) [I10–I15]	0	Kostev 2019			
	Cerebrovascular diseases [I60–I69]	0	Kostev 2019			
	Atherosclerosis [I70]	2	Kostev 2019	Kostev 2019		
	I24, I25 coronary heart disease	1	Kostev 2019	Kostev 2019		
	other and unspecified disorders of the circulatory system [I95–I99]	0	Kostev 2019			
	I95 hypotension	1	Kostev 2019	Kostev 2019		
	hemolytic anemias (nutritional anemias [D50–D53]	0	Kostev 2019			
	hemolytic anemias [D55–D59]	0	Kostev 2019			
	aplastic and other anemias [D60–D64]	0	Kostev 2019			

# = total number.
